# Educational Intervention among Barbers to Improve Their Knowledge regarding HIV/AIDS: A Pilot Study from a South Asian Country

**Published:** 2014-09

**Authors:** Mukesh Kumar Krishanani, Faridah Amir Ali, Ali Khan Khuwaja (Late), Waris Qidwai, Badar Sabir Ali

**Affiliations:** ^1^Saudi Postgraduate Family Medicine Program, Al-Khobar, Eastern Province, Saudi Arabia; ^2^Department of Family Medicine, Aga Khan University, Karachi, Pakistan; ^3^Department of Community Health Sciences, Aga Khan University, Karachi, Pakistan

**Keywords:** Acquired immunodeficiency syndrome, AIDS, Barbers, Educational intervention, Human immunodeficiency virus, HIV, Pakistan

## Abstract

One of the Millennium Development Goals is to combat HIV, the burden of which continues to increase in developing countries, like Pakistan. The prevalence is high among the high-risk population, and the use of unsterilized surgical instruments, traditional straight razors, and blades adds to the spread of this disease. This study assesses the effect of an educational intervention on the knowledge of 70 barbers practising in a suburban community in Pakistan regarding HIV and its symptoms and transmission. At baseline, only 10% of the barbers reported that they had ever heard about HIV compared to 49% after the intervention. Similarly, 4% and 6% of them had good knowledge at baseline about symptoms and transmission of the disease, increasing to 39% and 43% respectively, after the intervention (p<0.001). The results of this educational intervention warrant consideration of activation of mass campaigns to increase public awareness about bloodborne diseases and to educate personnel who might harm the persons in their communities by unsafe practices.

## INTRODUCTION

Combating human immunodeficiency virus (HIV) is one of the Millennium Development Goals (1), and illnesses associated with acquired immunodeficiency syndrome (AIDS) are still one of the leading causes of mortality at early age around the world (1). It is reported that 2.7 million new persons got infected with HIV globally in 2008, out of whom 350,000 were from Asia (1). India already comprises half of Asia's HIV prevalence while its burden continues to increase in other countries of South Asia, including Bangladesh and Pakistan (1).

Unsafe injections account for 3% of the new HIV cases (2). Besides the risk from injections, the risk of HIV from illicit drugs and the use of unsterilized sharp objects, such as straight razors and blades, also pose a potential risk for HIV infection (3-5). Surgical procedures, such as circumcision with unsterilized instruments—needles and blades—by traditional customs can spread HIV (6). Sharing a common razor at the barber's shop also carries a potential risk for spreading HIV (7). It is commonly observed that, in Pakistan, many barbers at their salons use traditional straight razor which is also known as ‘open razor’ or ‘cut-throat razor’ (5). At its one end, the half of the blade is fixed to use for shaving. With a high prevalence of roadside barbers shaving with unsterilized and re-used straight razors and blades (5,7,8), the practices of barbers in South Asia region may contribute to the spread of diseases, like hepatitis B, hepatitis C, and HIV. This is mainly due to illiteracy and unawareness about transmission of these infectious diseases (7,8).

A study from a developed country reported 93% awareness among hairdressers about HIV, viral hepatitis, and their modes of transmission (9). In contrast, studies from a South Asian country found that more than 50% of the barbers were unaware about the importance of sterilizing equipment, particularly straight razors and blades, to prevent HIV transmission (7,8,10). Khandait *et al*. revealed that a large proportion of the roadside barbers in India were unaware of modes of transmission of HIV in India (7). Similar results have been reported in Pakistan where 58% of barbers were unaware of any health hazards associated with their profession. However, 42% knew about hepatitis and AIDS (10). Some of the barbers also performed minor surgeries, like circumcision, excision of in-grown toe nail, and abscess drainage, which could be a procedure resulting in HIV/AIDS transmission (10).

Interventions have been done to improve the sexual and injection practices in South Asia (11). However, not much work has been done to improve the knowledge of barbers about HIV/AIDS.

Therefore, this study was done to assess the knowledge of barbers about HIV, followed by an educational intervention to determine if the knowledge of barbers in relation to HIV improved. This is the first study in Pakistan in which barbers were brought under an educational intervention to improve their knowledge regarding HIV/AIDS and its transmission.

## MATERIALS AND METHODS

After obtaining ethical approval from the institutional Ethical Review Committee of the Aga Khan University Hospital, this interventional study was conducted in 2007 in Karnal Basti which is an underprivileged area of Karachi. The majority of the population in this area belonged to the lower socioeconomic and lower educational class.

In Karnal Basti, there were 38 salons and 79 barbers who were working in these salons. At baseline, out of a sample of 79, a structured pretested questionnaire was administered among 70 barbers who gave consent. Nine barbers refused to participate, citing time constraint as a reason for not participating. The questionnaire included questions about sociodemographic characteristics and multiple-choice questions on the knowledge of these barbers about HIV/AIDS, its symptoms, transmission, and prevention. To maintain consistency, the sets of questionnaire were filled by the principal investigator (PI) himself. Filling of the baseline questionnaire was followed by a 30-min educational intervention which was again given by the PI. Pictorial and informative charts describing and illustrating the modes of transmission of HIV and prevention were developed and used for intervention after extensive review by the study investigators and other experts in this field. Further, a presentation was given to the participants communicating the modes of transmission and risk factors of HIV. The presentation was followed by a question-and-answer session in which the participants were encouraged to ask questions relevant to the subject. Finally, the barbers were requested to display educational material in their salons. This intervention was repeated twice—one month and two months after the initial intervention. At 3 months, the barber's knowledge was assessed using the same questionnaire. Cutoff scores were set to assess the level of knowledge about HIV. A ‘correct’ answer was taken as 1 and an ‘incorrect’ answer or ‘not known’ answer was taken as 0. Out of the total maximum score of 6, a score of ≥4 indicated ‘good’ knowledge about spread of HIV. Similarly, correct knowledge about symptoms of HIV was assigned a score of 1, and incorrect response was assigned a score of 0; out of the total maximum score of 4, a score of ≥2 denoted ‘good’ knowledge about symptoms of the disease. A change in knowledge after the intervention was assessed. Data were analyzed using Statistical Package for Social Sciences (SPSS) (version 17.0). The McNemar test was used in identifying the difference between individual responses, before and after the intervention. Fisher's exact test was used for overall score, if the cell counts were below 5. A p value <0.05 was considered to be significant for a change in knowledge after the intervention.

## RESULTS

Of the 70 participants, all completed the baseline survey, attended both educational sessions, and completed the post-intervention questionnaire. All barbers who participated were male, and most of them had no schooling (95%) or only up to 5 years of schooling. The mean age of participants was 24.3±9 years, with a range of 11-50 years while the mean duration of practice was 8.5±5.6 years. The mean number of clients that the barbers had for hair cutting, shaving faces, and/or armpits each day was 8±3.2.

Comparison of knowledge of barbers about HIV in the pre- and post-intervention period, is given in the table. Only 10% of the barbers had ever heard about HIV before the intervention compared to 49% following the intervention. Only 6% of the study participants said they knew how HIV could be prevented at baseline compared to 37% after the intervention.

Responses of barbers about modes of spread are mentioned in the table. Similarly, two variables about symptoms of HIV are mentioned. Other symptoms asked for were on lymphadenopathy and asymptomatic ones (p values for change in knowledge was insignificant). Applying the McNemar test for statistical significance, the differences in knowledge before and after the intervention about symptoms (p<0.001) and transmission of HIV (p<0.001) were significantly different, indicating an improvement in the knowledge.

**Table.** Knowledge of barbers in Karnal Basti, Pakistan, regarding HIV, its transmission and symptoms before and after an educational intervention, 2007

Variable	Correct response (%)		
	Pre-intervention	Post-intervention	p value
Knowledge of HIV			
Do you know what HIV is?	10	49	<0.001
Is HIV a disease of the immune system?	1	26	<0.001
Is there any vaccine to prevent HIV?	96	89	0.180
Modes of transmission of HIV			
Can HIV spread by blood transfusion?	3	33	<0.001
Can HIV spread by re-used blades?	6	39	<0.001
Can HIV spread by re-used syringes?	3	41	<0.001
Can HIV spread from mother to child?	1	30	<0.001
Can HIV spread by unprotected sex?	3	43	<0.001
Can HIV spread by shaking hands?	2	1	1.00
Good knowledge about spread (score of ≥4)	4	39	<0.001
Symptoms of HIV			
Is fever a symptom of AIDS?	1	16	0.01
Is weight loss a symptom of AIDS?	1	3	0.5
Is lymphadenopathy a symptom of HIV?	1	1	1.00
Can HIV be asymptomatic?	0	1	1.00
Good knowledge about symptoms (score of ≥2)	6	43	<0.001

## DISCUSSION

In South Asian countries, the practice of shaving at a barber's shop, especially by roadside barbers who provide their services at a very low cost, is well-reported but is rarely addressed as a mode of transmission of bloodborne infections. Despite the advent of safety razors, the practice of utilizing barbers’ services for shaving beard and armpits has been reported to be as high as 50% (12). Barbers working on roadside or even in shops are usually non-literate and are unaware of the bloodborne infections which can be transmitted by their practices (10,13). The straight razors and re-used blades can get contaminated either by cuts caused during use or even the microtrauma that can occur during shaving. Although the evidence is not very clear, HIV can survive outside the body for days which is also influenced by virus titre and volume of blood (14). Not only can these instruments infect the barbers providing services and the clients availing barbering services but also the sweepers and garbage scavengers who are usually children looking for valuables in the wastes, with bare hands (8). The United Nations Joint Programme on HIV/AIDS (UNAIDS) reports that Pakistan is a high-risk country, with low prevalence of the spread of HIV (2). Preventive strategies should be the mainstay; therefore, non-sexual transmission mechanism, including re-use of injection and potentially contaminated material that are most important mode of spread, warrants attention not only to prevent unsafe injectable drugs but also educating the personnel who can transmit these infections via use of unsterile contaminated equipment (13).

Educating barbers can help them improve their own knowledge, attitudes, and practices. In addition to this, team of same barbers could also be used for increasing awareness among their own community to increase the effectiveness of the health education programme. This could be a step towards risk reduction and, hence, disease prevention. Our study shows that barbers can be trained to improve their own knowledge and, thus, may be able to modify their practices for prevention of bloodborne infectious diseases, such as HIV.

The results of our study showed that, at baseline, 90% of the barbers had not even heard about HIV/AIDS. Majority of those who had heard about it were aware of its mode of transmission, although the number of such participants was not large. This is inconsistent with findings from the developed world where majority of practising barbers have knowledge about these bloodborne infections and their transmission (9). This is also less than that reported by Wazir *et al*. (10) who showed that 42% of the barbers had at least heard about hepatitis and AIDS. This may be because barbers may be more aware of ‘hepatitis’ than ‘HIV’ as it is more common in our part of the world and the governments and NGOs are specifically putting efforts for mass vaccination for hepatitis B in endemic areas. Also, in the previous study, 58% of the barbers were non-literate compared to 95% in our study. This may be the reason for low understanding even after the intervention about the disease. Still, the knowledge improved significantly; after the educational intervention, half of the participants said that they are aware of the disease, and more than a quarter knew the mode of transmission and reported knowledge about its prevention. Interestingly, the knowledge about existence of HIV vaccine was reported more than the knowledge about HIV itself. This may be due to their prior knowledge about hepatitis and its prevention with vaccine, and they might still be confusing the two diseases. Considering that these barbers, especially those on the roadside, are poor and cannot afford fancy disinfection procedures (13), educating them to change blades between clients, which would additionally cost them only US$ 0.02, may not be very difficult as this cost can be included in the clients’ service charges. In addition, mass media campaigns that inform the public about these harmful practices and advise clients to request for a new or sterile instrument from a barber or carry their own shaving kits to the barber shops could be implemented.

### Limitations

This study has several limitations which should be considered. First, only one small area in Karachi was included so that all barbers in that region could be targeted but the number of participants was small. Second, the salon environment may not have been the ideal location for this interview but still the interviewer tried to make the interview as private as possible. Third, it represents the self-reported data; so, it may be subject to social desirability bias. Finally, the follow-up period of the evaluation was only 3 months. It would have been better to conduct sessions at 6 months and/or 12 months after the first intervention to assess the post-intervention knowledge in the real sense.

### Conclusions

The results of this educational intervention are sufficient to activate mass campaigns to increase public awareness about bloodborne diseases that can be transmitted from barbers’ shops and to educate personnel who can harm the health of their communities by unsafe practices.

## ACKNOWLEDGEMENTS

We are very much grateful to all of our study participants who agreed to actively participate in the study in an underprivileged country. We are especially grateful to late Dr. Ali Khan Khuwaja; it was his thorough guidance which made us able to consolidate and publish the findings of this study.
